# Comprehensive knowledge about HIV/AIDS and associated factors among women of reproductive age in sub-Saharan Africa: a multilevel analysis using the most recent demographic and health survey of each country

**DOI:** 10.1186/s12879-022-07124-9

**Published:** 2022-02-07

**Authors:** Achamyeleh Birhanu Teshale, Yigizie Yeshaw, Adugnaw Zeleke Alem, Hiwotie Getaneh Ayalew, Alemneh Mekuriaw Liyew, Zemenu Tadesse Tessema, Getayeneh Antehunegn Tesema, Misganaw Gebrie Worku, Tesfa Sewunet Alamneh

**Affiliations:** 1 Department of Epidemiology and Biostatistics, Institute of Public Health, College of Medicine and Health Sciences, University of Gondar, Gondar, Ethiopia; 2 Department of Physiology, School of Medicine, College of Medicine and Health Sciences, University of Gondar, Gondar, Ethiopia; 3 Department of Midwifery, School of Nursing and Midwifery, College of Medicine and Health Sciences, Wollo University, Dessie, Ethiopia; 4 Department of Human Anatomy, College of Medicine and Health Science, School of Medicine, University of Gondar, Gondar, Ethiopia

**Keywords:** Comprehensive knowledge, HIV/AIDS, Sub-Saharan Africa

## Abstract

**Background:**

Women of reproductive age in sub-Saharan African (SSA) share the greatest burden of the HIV/AIDS epidemic. Comprehensive knowledge about HIV is seen as pivotal in combating the epidemic. Therefore, this study aimed to assess comprehensive knowledge about HIV/AIDS and associated factors among women of reproductive age in sub-Saharan Africa.

**Objective:**

To examine comprehensive knowledge about HIV/AIDS and associated factors among women of reproductive age in sub-Saharan Africa.

**Methods:**

We used the most recent SSA countries Demographic and Health Surveys (DHS) data. To assess comprehensive knowledge, a composite score of six separate questions (can get HIV by witchcraft or supernatural means, can reduce risk of getting HIV by using condoms during sex, reduce the risk of getting HIV by having one sex partner only, can get HIV from mosquito bites, can get HIV by sharing food with a person who has HIV/AIDS, and a healthy-looking person can have HIV) was used. Those who answered all six questions correctly were considered to have comprehensive knowledge. To assess the factors associated with comprehensive knowledge of HIV/AIDS, we used a multilevel binary logistic regression model since the data had hierarchical nature.

**Results:**

In this study, the comprehensive knowledge about HIV/AIDS was 38.56% (95% CI: 38.32, 38.75). Both individual and community-level factors were associated with comprehensive knowledge about HIV/AIDS. Among individual-level factors, older age, having primary and above educational level, being from wealthy households, contraceptive use, listening to the radio, and reading newspaper were associated with higher odds of comprehensive knowledge about HIV/AIDS. Being from urban areas and the Eastern African region were the community-level factors that were associated with higher odds of comprehensive knowledge about HIV/AIDS.

**Conclusion:**

The study found that comprehensive knowledge of HIV/AIDS is low. Individual and community-level factors were associated with comprehensive knowledge of HIV/AIDS. Therefore, giving special attention to those young women, women who had no formal education, those from poor socioeconomic status, and those who are from remote areas could decrease the epidemics of HIV/AIDS by increasing the comprehensive knowledge about HIV/AIDS. Besides, it is better to strengthen media campaigns regarding HIV/AIDS to increase comprehensive knowledge about HIV/AIDS.

## Background

The human immunodeficiency virus (HIV) is a global public health problem that takes the lives of about 33 million people. By the end of 2019, an estimated 38.0 million individuals were living with HIV, with 1.7 million new infections and 690,000 HIV-related deaths [1]. The vast majority of HIV-positive people are living in low- and middle-income countries, with the majority (68%) in sub-Saharan Africa (SSA) [2, 3]. Furthermore, in SSA, women of reproductive age are at greater risks of the pandemic [4].

The objectives of the 2030 Sustainable Development Goal 3 is attaining good health and well-being for all [5, 6]. However, the HIV/AIDS pandemic is a potential challenge to the achievement of these goals and remains the greatest cause of morbidity and mortality in low and middle-income countries [7–9].

Currently, the new HIV infection rate is high and different factors are associated with the risk of acquiring HIV infections. According to different studies conducted elsewhere, factors such as educational status, age, wealth status, media exposure, drug use, and consumption of alcohol are associated with acquiring HIV infections [10–13].

The other potential explanation for the occurrence of a higher new infection is due to low comprehensive knowledge about HIV/AIDS. Different scholars revealed that comprehensive knowledge about HIV is seen as pivotal in combating the epidemic [14–16]. Globally, women of reproductive age are at risk of acquiring HIV infection and only 30% of them have comprehensive knowledge about HIV/AIDS [4]. According to different scholars, the prevalence of comprehensive knowledge in Africa, particularly in sub-Saharan Africa is low, which ranges from 19.3% in Ethiopia to 48.9% in Burundi [14, 17, 18]. Different factors such as education, wealth status, place of residence, sex of household head, region, and media exposure are associated with comprehensive knowledge about HIV/AIDS [14, 19–21].

While there has been a progress towards the United Nations program on HIV/AIDS’ 90-90-90 targets for prevention and treatment, the targets are not achieved by 2020 [3]. Besides, low comprehensive knowledge about HIV/AIDS is reported in different SSA countries and evidence regarding the factors associated with comprehensive knowledge about HIV remains scanty. Moreover, up to our knowledge, there was no study on comprehensive knowledge about HIV/AIDS by pooling data of sub-Saharan Africa countries. Therefore, we aimed to investigate comprehensive knowledge about HIV/AIDS among women of reproductive age and its associated factors in sub-Saharan Africa. This helps policymakers to prevent the epidemics of HIV/AIDS through increasing awareness about HIV/AIDS by giving priority to the identified vulnerable groups.

## Methods

### Data source

We have used the most recent SSA countries Demographic and Health Surveys (DHS) data. For each countries DHS, the most recent Population and Housing Census was used as a sampling frame. The DHS sample was stratified and selected in two stages and the survey target groups were women and men of reproductive age in randomly selected households of each country. Then, detailed information was collected on background characteristics, maternal and child health, HIV/AIDS, domestic violence, and other important public health problems. Five questionnaires were used to collect the DHS data: the household questionnaire, the woman’s questionnaire, the man’s questionnaire, the biomarker questionnaire, and the health facility questionnaire. The data collection tool was pretested and extensive training was given for the data collectors. Further information about DHS data collection technique, in general, the DHS methodology can be found in each countries survey report.

For this study, we used the most recent DHS data that was conducted from 2015 to 2020. There were 19 countries with DHS conducted in the study period. However, we appended 15 countries’ DHS data for our analysis since the four countries (Senegal, South Africa, Tanzania, and Angola) DHS had no observation regarding comprehensive knowledge towards HIV/AIDS (Table 1).

**Table 1:** Overall sample size and sample per each countries DHS and survey year

Country and region	Year	Total population (N = 202,270)	Percentage (%)
Eastern African region		86,188	41.61
Burundi	2016/17	16,468	8.14
Ethiopia	2016	14,599	7.22
Rwanda	2015	13,428	6.64
Uganda	2016	18,435	9.11
Zambia	2018/19	13,394	6.62
Zimbabwe	2015	9864	4.88
Western African region		98,076	48.49
Benin	2017/18	7053	3.49
Gambia	2019/20	11,575	5.72
Guinea	2018	8811	4.36
Liberia	2019/20	7622	2.89
Mali	2018	8979	4.44
Nigeria	2018	39,433	19.50
Sierra Leone	2019	14,603	7.22
Central African region		18,006	8.90
Cameroon	2018/19	13,250	6.55
Chad	2015	4756	2.35

### Variables of the study

#### Outcome variable

The outcome variable in this study was comprehensive knowledge about HIV/AIDS. It was a composite score of six different questions: 1. Can get HIV by witchcraft or supernatural means, 2. Can reduce risk of getting through using condoms during sex, 3. Reduce the risk of getting HIV by having one sex partner only, 4. Can get HIV from mosquito bites, 5. Can get HIV by sharing food with a person who has HIV/AIDS, and 6. A healthy-looking person can have HIV [19, 22, 23].

Then a woman had correct comprehensive knowledge if she answers all the six questions correctly (said “No” for questions 1, 4, and 5 and said “Yes” for other questions) and not knowledgeable if she did not give the correct answer for at least one of the questions.

#### Independent variables

After searching literature [19–21, 23, 24], we have incorporated both individual and community level independent variables.

*Individual-level variables* maternal age, marital status, educational level, wealth status, sex of household head, contraceptive usage, reading a newspaper, listening to the radio, and watching television were the individual-level variables. Maternal age was categorized as 15–19, 20–24, 25–29, 30–34, 35–39, 40–44, and 45–49 years while marital status was categorized as single, married, widowed, and divorced/separated. The other variables were categorized as follows: educational level (no formal education, primary education, secondary education, higher education), wealth status (poorest, poorer, middle, richer, richest), sex of household head (male, female), contraceptive usage (yes, no), reading a newspaper (yes, no), listening to the radio (yes, no), and watching television (yes, no).

*Community-level variables* Place of residence and African region were incorporated as community-level variables. The place of residence was categorized as urban and rural. According to different literatures, African regions are categorized as Eastern, Western, Central, and Southern regions [25]. However, for our study, we did not have countries in the southern African region that have data on the outcome variable and, therefore, African regions were categorized as Eastern, Western, and Central African regions.

### Data management and statistical analysis

We used Stata version 14.0 software to extract, recode, and conduct the overall analysis. Throughout the analysis, we have applied weighting to restore the representativeness and to get a better statistical estimate (robust standard error) [26]. Due to the nature of the DHS data, we have done a multilevel analysis. Four multilevel models were fitted. The first model (model I) was fitted with only the outcome variable to assess the variability of the comprehensive knowledge about HIV/AIDS between clusters or to assess the intra-class correlation coefficient (ICC). The second model (model 2) was fitted using individual-level variables only. Model III was fitted with community-level variables only and model IV was fitted with both individual and community-level variables.

To assess the community level variability of comprehensive knowledge about HIV/AIDS (for random effect analysis), ICC, Median Odds Ratio (MOR), and proportional change in variance (PCV) were calculated. To verify model fitness, deviance was used and the best-fit model has been deemed a model with the lowest deviance.

Moreover, eligible variables for the multivariable analysis were selected using a bivariable analysis, and variables with a p-value < 0.20 in the bivariable analysis were qualified for the multivariable analysis. In the multivariable analysis, the adjusted odds ratio (AOR) with its 95% confidence interval (CI) was reported, and variables with a p-value < 0.05 were considered as significant predictors of comprehensive knowledge about HIV/AIDS.

## Results

### Sociodemographic characteristics of respondents

We used a total weighted sample of 202,270 women of reproductive age for this study. The majority (21.11%) of the respondents were in the age group 15–19 years. Most, 74.15%, of the respondents were from male-headed households and around 63.36% of the respondents were married. Regarding reading a newspaper and listening to the radio, about 16.92% and 57.39% of study participants read the newspaper and listens to the radio respectively. Moreover, the majority, 60.25% and 48.49% of study participants were from rural areas and the West African region respectively (Table 2).

**Table 2:** Sociodemographic characteristics of respondents

Characteristics	Weighted frequency (N = 202,270)	Percentage (%)
Age		
15–19	42,696	21.11
20–24	36,617	18.10
25–29	35,781	17.69
30–34	29,320	14.50
35–39	25,055	12.39
40–44	18,085	8.94
45–49	14,716	7.28
Educational level		
No formal education	63,081	31.19
Primary education	58,924	29.13
Secondary education	67,403	33.32
Higher education	12,862	6.36
Wealth status		
Poorest	34,095	16.86
Poorer	36,947	18.27
Middle	38,426	19.00
Richer	43,227	21.37
Richest	49,575	24.51
Sex of household head		
Male	149,979	74.15
Female	52,291	25.85
Contraceptive use		
Yes	46,196	22.84
No	156,074	77.16
Marital status		
Never in union	57,645	28.50
Married	128,166	63.36
Widowed	5488	2.71
Divorced/separated	10,971	5.42
Reading newspaper		
No	168,036	83.08
Yes	34,234	16.92
Listening radio		
No	86,180	42.61
Yes	116,090	57.39
Watching television		
No	114,050	56.38
Yes	88,220	43.62
Place of residence		
Urban	80,397	39.75
Rural	121,873	60.25
African region		
Eastern African region	86,188	41.61
Western African region	98,076	48.49
Central African region	18,006	8.90

### Comprehensive knowledge about HIV/AIDS in sub-Saharan Africa

**Fig. 1:**
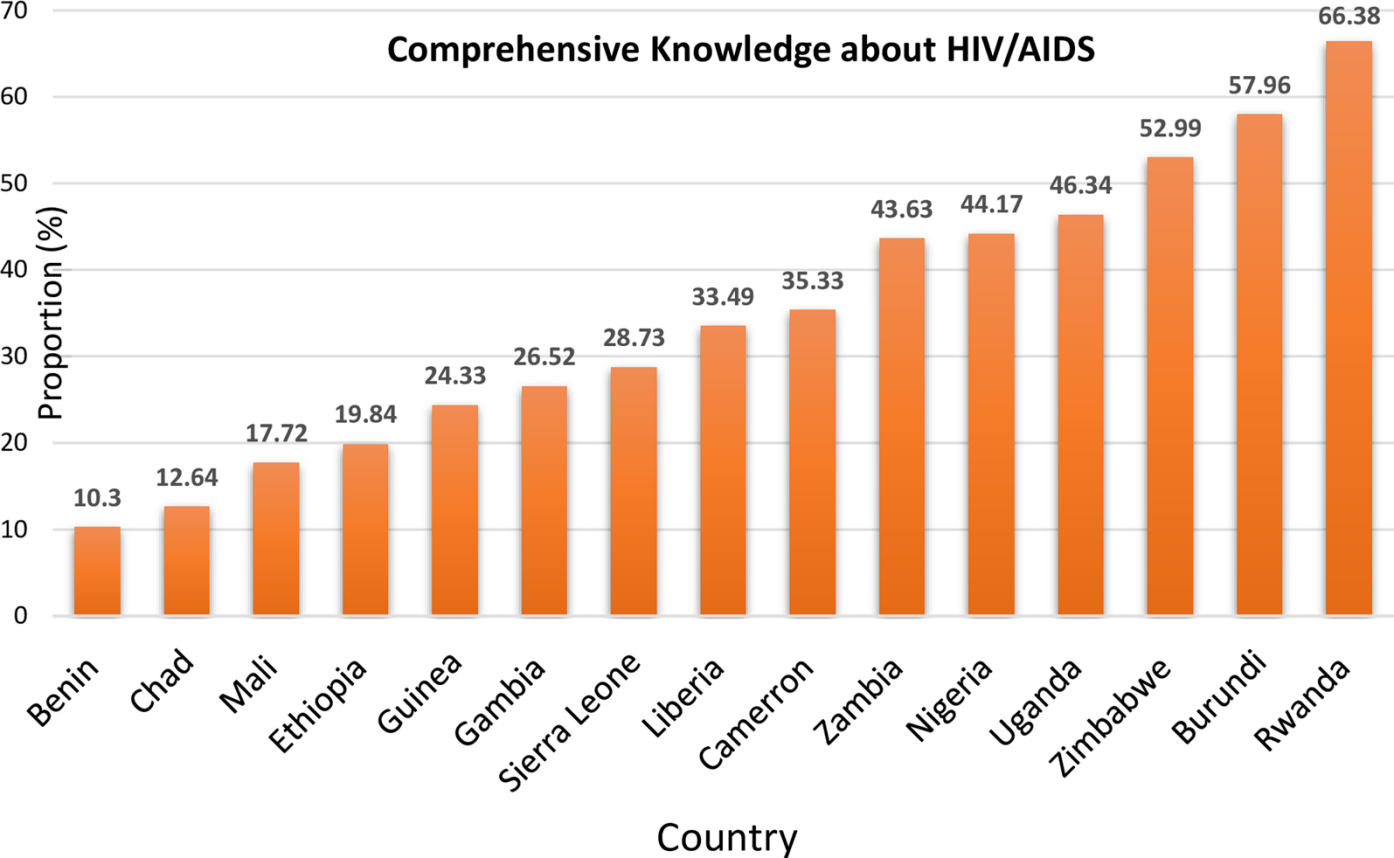
Comprehensive knowledge about HIV/AIDS by individual countries in sub-Saharan Africa

The comprehensive knowledge of HIV/AIDS was 38.54% (95% CI: 38.32, 38.75). The majority of the study participants had higher knowledge regarding each of the individual knowledge-related questions (Table 3). As illustrated in Fig. 1, there were wide differences in comprehensive knowledge about HIV/AIDS between individual countries, ranging from 10.3% (95% CI: 9.6, 11.03) in Benin to 66.38% (95% CI: 65.58, 67.17) in Rwanda (Fig. 1).

**Table 3:** Comprehensive knowledge of HIV/AIDS in sub-Saharan Africa

Variables	Frequency	Percentage (%)
1. Can get HIV by witchcraft or supernatural means		
Yes	38,755	19.16
No	163,515	80.84
2. Reduce the risk of getting HIV: always use condoms during sex		
No	44,381	21.94
Yes	157,889	78.06
3. Reduce the risk of getting HIV: have 1 sex partner only		
No	23,894	11.81
Yes	178,376	88.19
4. Can get HIV from mosquito bites		
Yes	61,387	30.35
No	140,883	69.65
5. Can get HIV by sharing food with a person who has AIDS		
Yes	45,805	22.65
No	156,465	77.35
6. A healthy-looking person can have HIV		
No	41,279	20.41
Yes	160,991	79.59
7. Comprehensive knowledge		
No	124,323	61.46
Yes	77,947	38.56

**Fig. 2:**
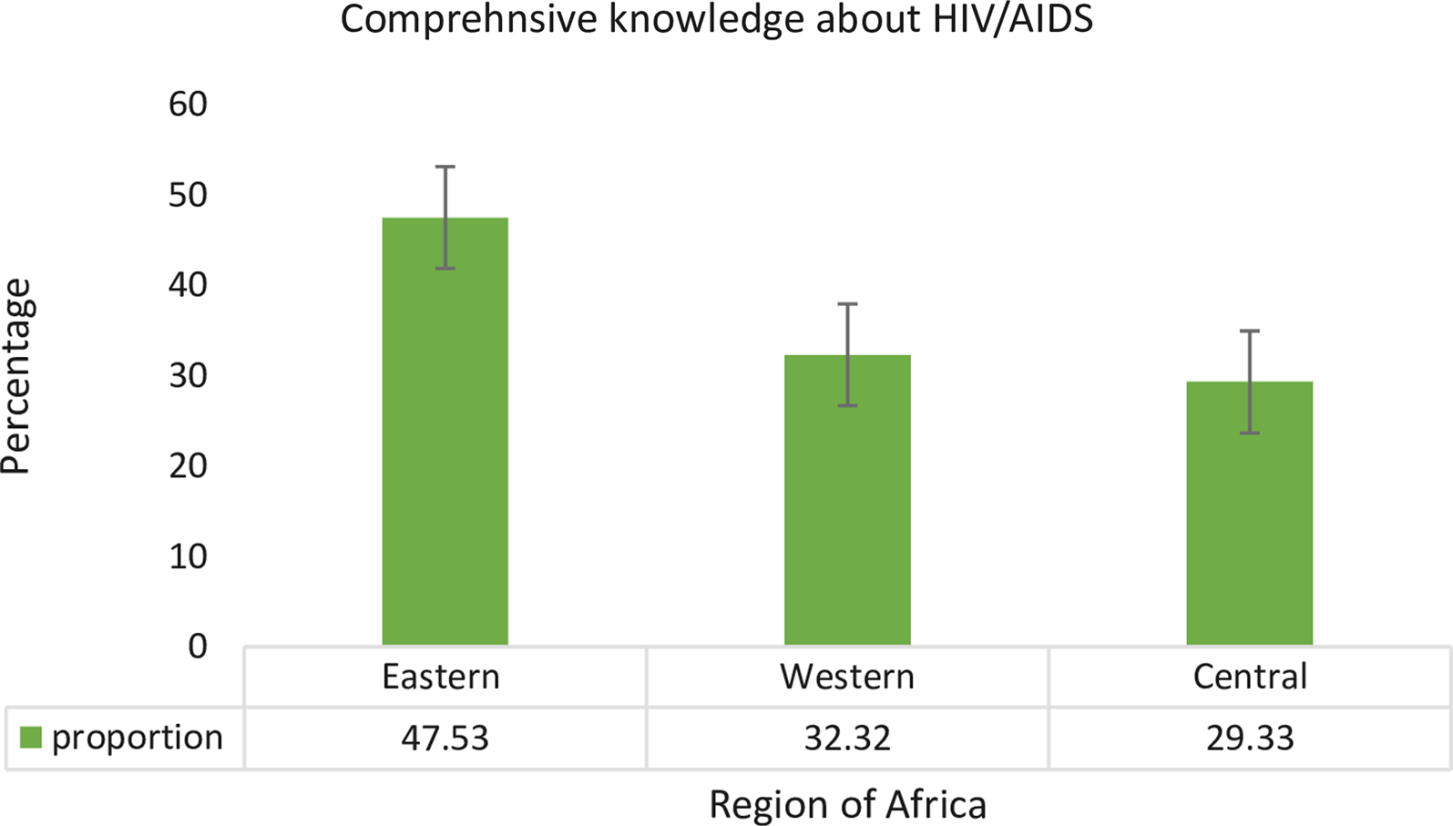
Comprehensive knowledge about HIV/AIDS by Africa regions

Moreover, as shown in Fig. 2, the comprehensive knowledge about HIV/AIDS was highest in the Eastern African region, which was 47.53% (95% CI: 47.20, 47.87) (Fig. 2).

### Factors associated with comprehensive knowledge about HIV/AIDS among women of reproductive age in sub-Saharan Africa

#### Random effect analysis

As we have seen from Table 4, all of the parameters favor the final model as the best model. The ICC in the model I indicates that about 10% of the variability in comprehensive knowledge about HIV/AIDS was attributed to the community/cluster level variability. The MOR in the model I also revealed that if we took two women from two different clusters (one from a cluster with higher comprehensive knowledge and one from a cluster with lower comprehensive knowledge) the odds of having comprehensive knowledge about HIV/AIDS among women who come from clusters with higher comprehensive knowledge was 1.78 times higher as compared to their counterparts. Moreover, the PCV in the final model (model IV) showed that about 24% of the variability in comprehensive knowledge was explained by both community-level and individual-level factors. Regarding model fitness, model IV was the best-fitted model since it had the lowest deviance and we used this model while assessing the factors associated with comprehensive knowledge about HIV/AIDS (Table 4).

**Table 4:** Random effect analysis for factors associated with comprehensive knowledge of HIV/AIDS among women of reproductive age in sub-Saharan Africa

Parameter	Model I	Model II	Model II	Model IV
Community-level variance	0.367	0.333	0.299	0.278
ICC	0.10	0.09	0.08	0.07
MOR	1.78	1.73	1.68	1.65
PCV	Reference	0.09	0.19	0.24
Log-likelihood	− 132,811.61	− 126,497.01	− 128,170.14	− 124,139.5
Deviance	265,623.22	252,994.02	256,340.28	248,279

#### Fixed effects analysis

All variables in the bivariable analysis were eligible for the multivariable analysis. In the multivariable multilevel analysis.

In the multivariable analysis, the odds of having comprehensive knowledge about HIV/AIDS was higher among older age groups as compared with those whose age was between 15 and 19 years. Being having a formal education, secondary education, and higher education, respectively, was associated with 1.37 (AOR: 1.37; 95% CI: 1.32, 1.44), 2.19 (AOR: 2.19; 95% CI: 2.08, 2.30), and 3.67 (AOR: 3.67; 95% CI: 3.42, 3.94) times higher odds of comprehensive knowledge as compared to those who had no formal education. Women who were from the middle, richer, and richest households had 1.14 (AOR: 1.14; 95% CI: 1.07, 1.20), 1.16 (AOR: 1.16; 95% CI: 1.09, 1.23), and 1.28 (AOR: 1.28; 95% CI: 1.19, 1.37) times higher odds of comprehensive knowledge, respectively, as compared to those who came from poorest households. Being using contraceptive methods was associated with 1.09 (AOR: 1.09; 95% CI: 1.05, 1.13) times higher odds of comprehensive knowledge as compared to their counterparts. Regarding reading newspaper, women who read the newspaper had 1.18 (AOR: 1.18; 95% CI: 1.13, 1.22) times higher odds of comprehensive knowledge compared to their counterparts. The odds of having comprehensive knowledge about HIV/AIDS was 20% (AOR: 0.80; 95% CI: 0.77, 0.82) lower among women who did not listen to the radio as compared with their counterparts. Being from the urban area had 1.10 (AOR: 1.10; 95% CI: 1.03, 1.18) times higher odds of comprehensive knowledge compared to their counterparts. Regarding the African region, women from the Eastern African region had 2.22 (AOR: 2.22; 95% CI: 2.09, 2.35) times higher odds of comprehensive knowledge about HIV/AIDS as compared to women from the Western African region (Table 5).

**Table 5:** Multilevel analysis of factors associated with comprehensive knowledge of HIV/AIDS among women of reproductive age in SSA

Variables	Model I	Model II AOR (95% CI)	Model III AOR (95% CI)	Model IV AOR (95% CI)
Age				
15–19		1.00		1.00
20–24		1.34 (1.29, 1.40)		1.33 (1.28, 1.38)***
25–29		1.47 (1.40, 1.53)		1.46 (1.40, 1.53)***
30–34		1.66 (1.58, 1.75)		1.63 (1.55, 1.72)***
35–39		1.61 (1.52, 1.69)		1.60 (1.52, 1.69)***
40–44		1.59 (1.50, 1.68)		1.56 (1.48, 1.65)***
45–49		1.58 (1.49, 1.68)		1.58 (1.48, 1.68)***
Educational level				
No formal education		1.00		1.00
Primary education		1.79 (1.72, 1.87)		1.37 (1.32, 1.44)***
Secondary education		2.37 (2.26, 2.49)		2.19 (2.08, 2.30)***
Higher education		3.78 (3.53, 4.06)		3.67 (3.42, 3.94)***
Wealth status				
Poorest		1.00		1.00
Poorer		1.03 (0.98, 1.08)		1.05 (0.99, 1.10)
Middle		1.13 (1.07, 1.19)		1.14 (1.07, 1.20)***
Richer		1.21 (1.14, 1.28)		1.16(1.09, 1.23)***
Richest		1.44 (1.35, 1.54)		1.28 (1.19, 1.37)***
Sex of household head				
Male		1.00		1.00
Female		1.03 (0.99, 1.06)		0.99 (0.95, 1.02)
Contraceptive use				
Yes		1.22 (1.18, 1.26)		1.09 (1.05, 1.13)***
No		1.00		1.00
Marital status				
Never in union		1.09 (1.04, 1.14)		1.04 (0.99, 1.09)
Married		1.00		1.00
Widowed		1.17 (1.09, 1.27)		1.08 (0.99, 1.17)
Divorced/Separated		1.09 (1.03, 1.16)		0.98 (0.93, 1.04)
Reading newspaper				
No		1.00		1.00
Yes		1.32 (1.27, 1.38)		1.18 (1.13, 1.22)***
Listening radio				
No		0.77 (0.74, 0.79)		0.80 (0.77, 0.82)***
Yes		1.00		1.00
Watching television				
No		1.00		1.00
Yes		0.80 (0.77, 0.84)		0.96 (0.92, 1.01)
Place of residence				
Urban			2.51 (2.07, 3.04)	1.10 (1.03, 1.18)**
Rural			1.00	1.00
African region				
Eastern African region			4.49 (3.61, 5.59)	2.22 (2.09, 2.35)***
Western African region			1.00	1.00
Central African region			0.85 (0.69, 1.05)	0.98 (0.89, 1.09)

***p value < 0.001, **p value < 0.01

## Discussion

This study found that the comprehensive knowledge about HIV/AIDS among women of reproductive age was 38.54%. Besides, there was a huge differences in comprehensive knowledge about HIV/AIDS between individual SSA countries, from 10.3% in Benin to 66.38% in Rwanda.

The proportion of comprehensive knowledge about HIV/AIDS found in this study, is consistent with a study conducted in Uganda [18] and lower than a study finding from Burundi, Kenya, and Uganda [14]. Besides, this figure is higher than findings from northern Uganda and Ethiopia [14, 27]. The variation found in this study (between SSA countries) and the variation with other previous studies may be due to the difference in socio-economic and socio-cultural characteristics of respondents between countries. In addition, this study is based on pooled analysis that incorporates data of the sub-Saharan African countries; others incorporate data of a single country.

In this study, different factors were associated with comprehensive knowledge about HIV/AIDS. The odds of having comprehensive knowledge about HIV/AIDS was higher among older women as compared to younger-aged women. This is in line with studies conducted in Bangladesh and Uganda [18, 28]. This may be because the traditional social system and health care service often bother older age group women. Besides, younger age women had a barrier to communicate with adults regarding sex-related information and sexually transmitted diseases.

Educational status had significantly associated with comprehensive knowledge about HIV/AIDS. Women with primary and higher educational status had higher odds of having comprehensive knowledge about HIV/AIDS compared to those mothers with no formal education. This finding is congruent with studies done in Ethiopia [19, 29], Bangladesh [24, 30], and Vietnam [31]. This may be since educated women can attain more knowledge when they are exposed to different information sources such as printed paper and radio. The other plausible explanation is education causes women to be more positive about their health and to look for information to protect themselves against HIV/AIDS. Moreover, educated women are more likely to get information regarding HIV from school-based HIV/AIDS interventions.

The study at hand revealed that mothers from the middle, richer, and richest households had higher odds of comprehensive knowledge about HIV/AIDS as compared to those who were from the poorest households. This is in agreement with studies conducted elsewhere [14, 18, 20, 21]. The possible explanation is having good socioeconomic status helps to accesses different media and increases educational achievement, which increases the likelihood of knowledge about HIV/AIDS [32].

Consistent with other studies conducted elsewhere [18, 19, 24, 28, 29], in this study, being having exposure to radio and newspaper was associated with higher odds of comprehensive knowledge about HIV/AIDS as compared to their counterparts. This might be since media has a huge influence in educating and conveying proper knowledge that reduces pre-existing misunderstandings regarding HIV/AIDS.

The study also identified women from urban areas had higher odds of comprehensive knowledge about HIV/AIDS as compared to women from rural areas. This is consistent with a study conducted elsewhere [18, 20, 28]. The possible reason could be rural women are often with the great problem in terms of access to health-related information, schooling, media, and healthcare facilities. Besides, those women who reside in rural areas also had less exposure to HIV/AIDS-related information such as HIV testing and counseling campaigns and different training sessions that increase awareness about HIV/AIDS.

Moreover, there were regional variations regarding comprehensive knowledge about HIV/AIDS in which women from the Eastern African region had higher odds of comprehensive knowledge about HIV/AIDS compared to those from the Western African region. This regional variation is supported by studies conducted in Bangladesh [28] and Ghana [20]. This could be due to the difference in terms of access to education, media, and sociocultural and socioeconomic status between regions.

This study had both strengths and limitations. It was based on a relatively large dataset. It was also based on an appropriate model (multilevel modeling) to account for the hierarchical nature of the DHS data. Despite that, our study had few important limitations. Due to the nature of data (secondary data), we had no control over confounders and the measurement of indicators. Since DHS data did not have country-level factors we did not consider them in the analysis.

## Conclusion

The study found that comprehensive knowledge about HIV/AIDS in sub-Saharan Africa is low. Factors both at the individual and community level were associated with comprehensive knowledge about HIV/AIDS. Therefore, giving special attention to those young women, women who had no formal education, those from poor socioeconomic status, and those who are from remote areas could decrease the epidemics of HIV/AIDS by increasing the comprehensive knowledge about HIV/AIDS. Besides, it is better to strengthen media campaigns regarding HIV/AIDS to increase comprehensive knowledge about HIV/AIDS.

## Data Availability

All result-based data is in the manuscript and anyone can access the data set from https://dhsprogram.com.

## Abbreviations

AIDS: Acquired immunodeficiency virus
AOR: Adjusted odds ratio
CI: Confidence interval
HIV: Human immunodeficiency virus
ICC: Intraclass correlation coefficient
MOR: Median odds ratio
PCV: Proportionate change in variance
SSA: Sub-Saharan African
